# Resident Doctors’ Perspectives and Experiences with Spiritual/Religious Considerations: A Survey-Based Project

**DOI:** 10.1192/bjo.2025.10324

**Published:** 2025-06-20

**Authors:** Safia Zaffarullah, Tom Robson, Neelima Reddi

**Affiliations:** Surrey and Borders Partnership NHS Foundation Trust, Surrey, United Kingdom

## Abstract

**Aims:** The RCPsych’s position statement recommends a spirituality/religious belief (S/R) history be routinely considered in clinical assessments, and that an understanding of S/R and their relationship to psychiatric disorders are essential components of psychiatric training. In reality, psychiatric training at local and collegiate level is likely lacking in both quality and quantity of training in S/R considerations. There are likely a myriad of factors barring trainees from broaching this topic, which this survey-based QI project sought to explore.

**Aims:** 1. Explore Resident Doctors’ attitudes towards the significance of spirituality/religion (S/R) in mental health. 2. Explore Resident Doctors’ experiences and satisfaction with S/R training in Psychiatry. 3. Explore current frequency with which Resident Doctors’ consider S/R in clinical assessments. 4. Establish Resident Doctors’ perceived confidence in and barriers to exploring S/R in their clinical assessments.

**Methods:** An online, self-completion questionnaire was constructed for a target population of 108 Resident Doctors employed with SABP trust during the survey window from 16/07/2024 to 27/08/2024. It was advertised through work email and WhatsApp groups.

The survey included 8 quantitative items and 1 free-text item, exploring various aspects of trainees’ perceptions around S/R. The quantitative items were analysed using simple descriptive statistics and the free-text item was analysed using thematic analysis via coding.

**Results:** The survey received a total of 39 responses (1 FY Doctor, 8 GP trainees, 19 core trainees and 11 higher specialty trainees).

Quantitative analysis: 90% felt S/R consideration was extremely or somewhat important clinically; 54% felt either neutral or not confident in exploring S/R clinically; 49% reported they rarely or never consider S/R clinically; 15% reported satisfaction with current S/R training; 90% felt there was an unmet training need on S/R considerations in Psychiatry.

Common barriers to consideration included: Lack of training (64%), concerns around triggering psychopathology (46%) and lack of time (46%).

Qualitative analysis: The free-text item explored any other perceived barriers. Responses were coded into 7 clear themes: fear of litigation, cultural barrier, lack of awareness, time pressures, lack of prompting and lack of training.

**Conclusion:** This project highlights a significant disconnect between the RCPsych’s position on spirituality and trainees’ current experiences. Interestingly, the common barriers that emerged are all factors that can be addressed via formal teaching/training at the local and regional level, which should seek to dispel fears and normalise conversation around and consideration of this oft neglected yet essential aspect of holistic mental health care.

